# Radioprotectant Activity of 5-Diethylsulfonamoylsalicylatocopper(II) in Gamma Irradiated Mice

**DOI:** 10.1155/MBD.1999.135

**Published:** 1999

**Authors:** John A. Kuykendall, III, Hugh Simmons, III, Henry J. Irving, Mark A. Wear, Paul G. Sorenson, Linda G. Tipton, Kenchasha M. Maddox, Elsie L. Williams, Van Anh Pham-Tran, Chassie Credit, Israt L. Chowdhury, Shaheen Khan, J. Brooks Tipton, William M. Willingham, John R. J. Sorenson

**Affiliations:** 1 Research Center University of Arkansas at Pine Bluff Pine Bluff Arkansas 71601 USA; 2 Department of Medicinal Chemistry College of Pharmacy University of Arkansas for Medical Sciences Campus Little Rock Arkansas 72205 USA

## Abstract

Survival and changes in mean body mass of whole-body irradiated mice were determined to examine the radioprotectant activity of 5-diethylsulfonamoylsalicylatocopper(II) [Cu(II) (5-DESS)]. One of four groups of 25 female C57BL/6 mice were treated subcutaneously (sc)with 0, 10, 20, 40, 60, 80, 100, or 120 μmol Cu(II)(5- DESS)/kg of body mass 3 hours before exposure to 8.0 Gy, gamma irradiation. In this paradigm, doses of Cu(II)(5- DESS) increased survival up to 92% above vehicle-treated control mice (P = 0.008). Mean body mass determinations revealed that mice treated with 80 to 120 μmol Cu(II)(5-DESS)/kg of body mass exhibited a smaller decrease in body mass than other complex-treated groups. These results support the hypothesis that Cu(II)(5-DESS) is an effective radioprotectant.


### RADIOPROTECTANT ACTIVITY OF 5-DIETHYLSULFONAMOYLSALICYLATOCOPPER(II) IN GAMMA IRRADIATED MICE

John A. Kuykendall[1], III, Hugh Simmons, III[1], Henry J. Irving[1], Mark A. Wear[2], Paul G. Sorenson[2], Linda G. Tipton[1], Kenchasha M. Maddox[2], Elsie L. Williams[1], Van Anh Pham-Tran[2], Chassie Credit[2], Israt L. Chowdhury[1], Shaheen Khan[1], J. Brooks Tipton[1], William M. Willingham[1], and John R.J. Sorenson*[2]

[1]Research Center, University of Arkansas at Pine Bluff, Pine Bluff, Arkansas 71601, USA
[2]Department of Medicinal Chemistry, College of Pharmacy, University of Arkansas for Medical Sciences Campus, Little Rock, Arkansas 72205, USA; e-mail: sorensonjohnr@exchange.uams.edu

ABSTRACT

Survival and changes in mean body mass of whole-body irradiated mice were determined to examine the radioprotectant activity of 5-diethylsulfonamoylsalicylatocopper(II) [Cu(II) (5-DESS)]. One of four groups of 25 female C57BL/6 mice were treated subcutaneously (sc) with 0, 10, 20, 40, 60, 80, 100, or 120 μmol Cu(II)(5-DESS)/kg of body mass 3 hours before exposure to 8.0 Gy, gamma irradiation. In this paradigm, doses of Cu(II)(5-DESS) increased survival up to 92% above vehicle-treated control mice ($P = 0.008$). Mean body mass determinations revealed that mice treated with 80 to 120 μmol Cu(II)(5-DESS)/kg of body mass exhibited a smaller decrease in body mass than other complex-treated groups. These results support the hypothesis that Cu(II)(5-DESS) is an effective radioprotectant.

INTRODUCTION

Protecting tissues from harmful effects of ionizing radiation or facilitating recovery from radiation injury are important in reducing injurious aspects of accidental, environmental, occupational, and therapeutic exposure to radiation. Agents used for these purposes are of particular importance with regard to facilitating manned space travel enabling prophylaxis or treatment of solar radiation injury, treatment of radiation injury due to exposure to radioactive material from electric power generating facilities, as well as gamma-ray, x-ray, and ultraviolet electromagnetic radiation generated by high voltage power distribution lines, radiotherapy of neoplastic tumors, and electromagnetic radiation due to the transparency of the ozone layer [1]. Radioprotector and radiorecovery agents, agents administered after radiation injury [2], are essential in planning the manned space program and decreasing the adverse health effects of intended and accidental exposure to radiation.

In 1942, Dale observed that addition of colloidal sulfur and thiourea to an aqueous solution of carboxypeptidase prior to x-irradiation protected the enzyme from inactivation [3]. Since this report, thousands of compounds have been screened for use as radioprotectants [4,5]. The best known and most widely studied of these compounds is S-2-(3-aminopropylamino)ethyl phosphorothiotic acid (WR-2721). However, WR-2721 is plagued by two major problems, which are associated with all aminothiol-based compounds, high toxicity and an inability to substantially increase survival when administered after irradiation [6].

The discovery that a cytostolic essential metalloelement-dependent enzyme, dicopper(II)dizinc(II) Superoxide Dismutase (CuZnSOD), increased survival of lethally irradiated mice when administered both one hour before and after irradiation [7], suggested a new approach to radiation protection. This new approach was consistent with the recognition that radiation injury is increased in the presence of oxygen and the formation of superoxide [8,9].

There is a rich history describing the occasional use of essential metalloelements, including Cu, as radioprotective agents [5,10]. Most recently, the radioprotective effect of dicopper tetrakis-(3,5-diisopropylsalicylate), [$Cu(II)_2(3,5\text{-DIPS})_4$]. [$Cu(II)_2(3,5\text{-DIPS})_4$] was examined based upon its SOD-mimetic activity [11]. It has subsequently been shown that $Cu(II)_2(3,5\text{-DIPS})_4$ is effective when given intraperitoneally, subcutaneously, or orally at nontoxic doses either before or after irradiation [12-14]. Doses of $Cu(II)_2(3,5\text{-DIPS})_4$ used in reported studies range from one-fifth to one-hundredth of the acutely toxic dose for male or female mice respectively [12].

Steel et al. [14] found that 24 to 48 μmol $Cu(II)_2(3,5\text{-DIPS})_4$/kg of body mass and 49 to 96 μmol $Cu(II)Cl_2$/kg of body mass given sc 24 hours prior to irradiation were radioprotective; while doses of 133 to 901

μmol 3,5-diisopropylsalicylic acid/kg of body mass produced no radiation protection. It is most likely that copper complexes formed *in vivo* following the administration of $Cu(II)Cl_2$ ultimately account for its radioprotective effects [2]. Use of complexes avoids irritation caused by injection of $Cu(II)Cl_2$or $Cu(II)SO_4$ due to their strong Lewis acidity and complexes are less acutely toxic than these inorganic forms of copper. Complexes also enable tissue targeting with regard to propitious distribution of essential metalloelements.

Since $Cu(II)_2(3,5\text{-DIPS})_4$ is a non-antigenic, SOD-mimetic complex [15] we addressed the question as to whether or not other non-antigenic, SOD-mimetic copper complexes might also be effective radioprotectants. Toward this end we studied the radioprotectant efficacy of another SOD-mimetic, copper(II) 5-diethylsulfonamoylsalicylate [Cu(II)(5-DESS)]. Its $IC_{50}$ in the xanthine/xanthine oxidase-nitroblue tetrazolium system was found to be 3.4 μM which is essentially the same as the $IC_{50}$of 1.5 μM found for $Cu(II)_2(3,5\text{-DIPS})_4$.

To examine the radioprotectant activity of Cu(II)(5-DESS), female C57BL/6 mice were treated subcutaneously (sc) with 0, 10, 20, 40, 60, 80, 100, or 120 μmol Cu(II)(5-DESS)/kg of body mass 3 hours before being exposed to 8.0 Gy gamma irradiation. Body mass measurements were also recorded daily throughout the 30 day post-irradiation period to examine the effect of Cu(II)(5-DESS) on radiation-induced loss of body mass.

### METHODS AND MATERIALS

5-Diethylsulfonamoylsalicylic acid (5-DESS) was synthesized as follows:
Five ml (0.048 moles) of diethylamine (Sigma) was placed in a 100 mL round bottom flask with a stirring bar and warmed to 50°C. 5-Chlorosulfonylsalicylic acid (2.47g, 0.01 mole) (Fischer Scientific) dissolved in 5 ml of Tetrahydrofuran (Aldrich), was then slowly added to the round bottom flask with stirring. Five ml of 1N NaOH (Mallinckrodt) was then added and the mixture heated at 50°C for 1 hr and then heated at 90°C for one-half hour. After cooling in an ice bath, concentrated HCl was added dropwise with stirring until the pH reached 2.0. The tetrahydrofuran was removed by flash evaporation and the filtered solid recrystallized from a 1:1 mixture of 95% ethanol and deionized water. The off-white solid melted through the range of 135°C to 136°C. Elemental analysis calculated for $C_{11}H_{15}NO_5S$: C; 48.35, H; 5.49, N; 5.12, and S; 11.72%. Found: C; 48.17, H; 5.50, N; 4.93, and S; 11.66%.

The copper complex of 5-DESS was synthesized by dissolving 0.2 g (0.73 mmol) of 5-DESS in 45 ml of 95% ethanol with 0.029 g (0.73 mmol) of aqueous NaOH in 15 ml of 95% ethanol. Cupric chloride dihydrate (Mallinckrodt) (0.07g, 0.37 mmol), dissolved in 15 ml of 95% ethanol was added dropwise with vigorous stirring . After this solution was concentrated to two-thirds of the final volume and refrigerated for 3 days, a pale-blue precipitate formed and was collected by filtration, washed with 95% ethanol, and dried at 40°C overnight. This complex did not melt on heating but decomposed over the range of 290°C to 301°C. Elemental analysis calculated for $C_{11}H_{13}NO_5SCu$: C; 37.43, H; 4.30, N; 3.97, O; 27.22, S; 9.10, and Cu; 18.00%. Found: C; 37.58, H; 4.50, N; 3.95, O; 27.09, S; 9.12, and Cu; 17.87%.

Propylene glycol (Sigma) was microbiologically sterilized by Millipore Filtration through a 0.2 μM filter. One liter of a 1.4% mass/volume polyvinyl alcohol (Sigma) was prepared by dissolving 14 grams in 900 ml saline at 90°C with stirring, cooling to room temperature, and diluting to 1000 ml after removal of the stirring bar.

Solutions of Cu(II)(5-DESS) were prepared as follows. The complex (23 mg, 64 μmol) was first wetted with 1.6 ml of propylene glycol in a glass Potter-Elvehjem homogenizer with a Teflon pestle. The resulting emerald green solution was transferred to a graduated cylinder with 1.4% polyvinyl alcohol in saline, used to rinse the homogenizer, to a total volume of 40 ml, and vortex stirred. This slow dilution produced a light-green colored true solution. A volume of 0.5 ml of this solution contained 0.8 μmol of complex and provided the 40 μmol/kg of body mass dose of Cu(II)(5-DESS). Ten or five ml of this solution was diluted with 10 or 15 ml of complete vehicle (4% propylene glycol and 1.4% polyvinyl alcohol in saline), respectively and vortex stirred to obtain solutions containing 20 or 10 μmol/kg of body mass dose per 0.5 ml of solution.

Solutions of 40, 60, or 80 μmol/kg of body mass were prepared by first wetting the complex (115 mg, 320 μmol) with 4 ml of propylene glycol in a 10 ml beaker. This solution was stirred and transferred to a graduated cylinder with 1.4% polyvinyl alcohol in saline to a total volume of 100 ml and vortex stirred. A volume of 0.5 ml contained 1.6 μmol of complex and provided 80 μmol Cu(II)(5-DESS)/kg of body mass. Fifteen ml or 10 ml of the 3.2 μmol per ml solution was diluted with 5 ml or 10 ml of complete vehicle to obtain solutions containing 1.2 μmol or 0.8 μmol per 0.5 ml of solution which provided 60 μmol or 40 μmol Cu(II)(5-DESS)/kg of body mass
Solutions of 1.6, 2.0, 2.4 μmol per 0.5 ml intended to administer 80, 100, 120 μmol Cu(II)(5-DESS)/kg of body mass were prepared by first wetting the complex (69 mg, 196 μmol) with 1.6 ml of propylene glycol in a 10 ml beaker and then the contents of the beaker transferred to a graduated cylinder and vortex stirred with enough 1.4% polyvinyl alcohol to make 40 ml of a 2.40 μmol per 0.5 ml of solution, providing 120 μmol Cu(II)(5-DESS)/kg of body mass.

Fifteen ml of this solution was then diluted with 3 ml of complete vehicle and vortex stirred to obtain 18 ml containing 2.0 μmol of Cu(II)(5-DESS) per 0.5 ml, providing a dose of 100 μmol/kg of body mass. Twelve ml of the 2.4 μmol per 0.5 ml solution was then diluted with 6 ml of complete vehicle and vortex stirred to obtain 18 ml of a 1.6 μmol per 0.5 ml of solution, providing a dose of 80 μmol/kg of body mass.

Single dose samples (0.5 ml) were placed in metal-free culture tubes prior to initiation of treatment and following treatment of each group. These samples were analyzed for copper by atomic absorption spectrophotometry using an IL model 155 spectrophotometer and established interference-free methods to determine the actual treatment dose. A diluted sample of Cation Cal, a synthetic plasma (American Hospital Supply) or a diluted solution of trace metals (Environmental Resource Associates, Cat #500), served as external standards. Found values were within the $\pm$5% range of the calculated value. This procedure is recommended to provide assurance that the actual dose given is the intended dose. Mice were whole-body irradiated using a J.L. Sheperd and Associated 143-45 Cesium-137 irradiator with an existing dose-rate of 1.43, 1.35, or 1.30 Gy/min.

All animal experiments were approved by the Institution's Animal Care and Use Committee which has an Animal Welfare Assurance on file with the Office of Protection from Research Risks.

For each experiment, one hundred female C57BL/6 mice, purchased from the National Cancer Institute, were randomly assigned to 20 Plexiglass cages and given food and water ad libitum. Four groups of 25 ten week old (15 to 20 g) mice were treated sc at the nape of the neck 3 hours before an approximate $LD_{50/30}$ (8.0 Gy) dose of gamma radiation. Survivor body mass was determined daily throughout the 30 day duration of each experiment. Mice that have not died by day 30 following irradiation will experience long term survival in having overcome the effects of irradiation Mean body masses for each treatment group were plotted without standard error bars for clarity.

The Fisher Exact Two-Tailed Test was used to statistically compare results obtained for vehicle-treated and complex-treated groups. Percent change in survival due to treatment was calculated as follows: percent survival of complex-treated group minus percent survival of the vehicle-treated group divided by the percent survival of the vehicle-treated group multiplied by 100 equals percent change in survival.

### RESULTS

As shown in Figure 1, treatment with 20 or 40 μmol Cu(II)(5-DESS)/kg of body mass caused a modest increase in survival. These small and not statistically significant increases in survival were 15% or 30%, respectively, greater than vehicle-treated mice. It is noteworthy that the duration of 100% survival following irradiation increased with increasing dose of complex. Repeating this study using doses of 40, 60, or 80 μmol/kg of body mass caused greater increases in survival (Figure 2) to 66%, 66%, or 111% (P=0.01) respectively above vehicle-treated mice. Treatment with doses of 80, 100, or 120 μmol Cu(II)(5-DESS)/kg of body mass increased survival however only the 120 μmol/kg dose produced a statistically significant (P=0.008) increase in survival (Figure 3) and again it is clear that the duration of 100% survival following irradiation increased with increasing dose of complex. These survival results are summarized in Table I.

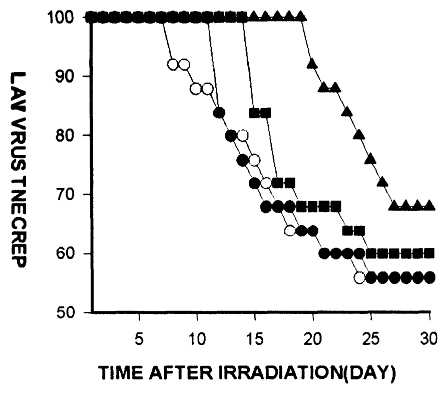

Figure 1. Survival of 8.0 Gy whole-body irradiated mice treated with vehicle (O), 10 μmol (●), 20 μmol (■), or 40 μmol (▲) Cu(II)(5-DESS)/kg of body mass.

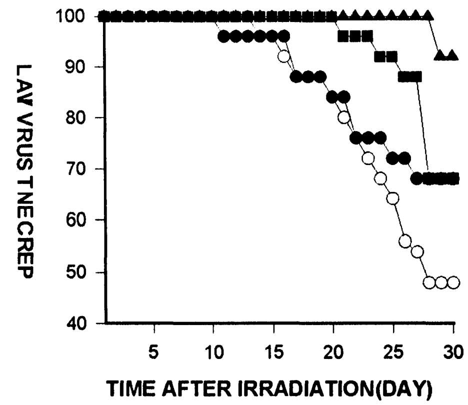

Figure 2. Survival of 8.0 Gy whole-body irradiated mice treated with vehicle (O), 40 μmol (●), 60 μmol (■), or 80 μmol (▲) Cu(II)(5-DESS)/kg of body mass.

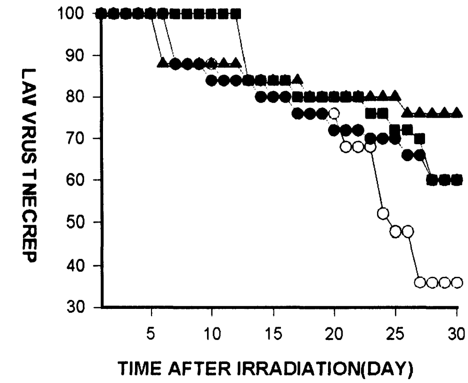

Figure 3. Survival of 8.0 Gy whole-body irradiated mice treated with vehicle (O), 80 μmol(●), 100 μmol (■), or 120 μmol (▲) Cu(II)(5-DESS)/kg of body mass.

**Table I. Radioprotectant Activities of Cu(II) (5-DESS)**

| Doses of Cu(II) 5-DESS (μmole/kg) | Percent Survival | Percent Increase | P Value |
|---|---|---|---|
| 0 | 52 | ----- | ----- |
| 10 | 52 | 0 | P=1.0 |
| 20 | 60 | 15 | P=0.78 |
| 40 | 68 | 30 | P=0.39 |
| 0 | 36 | ----- | ----- |
| 40 | 60 | 66 | P=0.16 |
| 60 | 60 | 66 | P=0.16 |
| 80 | 76 | 111 | P=0.019 |
| 0 | 48 | ----- | ----- |
| 80 | 68 | 42 | P=0.17 |
| 100 | 68 | 42 | P=0.17 |
| 120 | 92 | 92 | P=0.0008 |

As shown in Figure 4, 5, and 6, vehicle-treated and Cu(II)(5-DESS)-treated mice lost body mass after irradiation. Surviving mice recovered body mass by day 20 to 25. Mice treated with larger doses of 100 or 120 µmol Cu(II)(5-DESS) kg of body mass (Figure 6) appear to have had smaller losses in body mass following irradiation than any of the other treated mice. While there was very little difference in acute loss of body mass for complex-treated mice, the 40 and 60 µmol Cu(II)(5-DESS)/kg treated mice appear to have lost slightly but not sigmificantly more body mass than all other groups treated with Cu(II)(5-DESS).

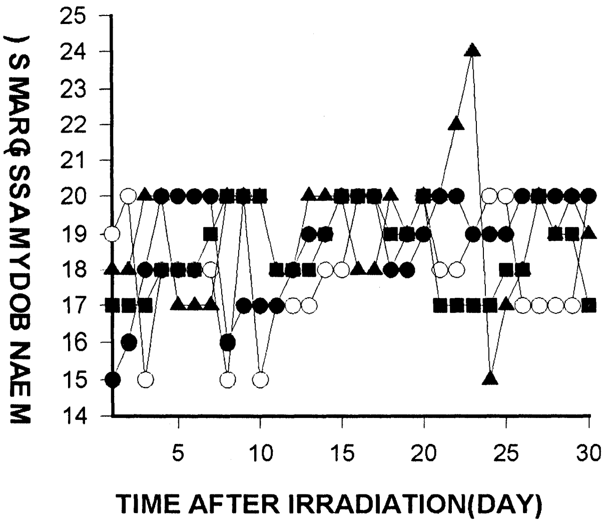

Figure 4. Mean body mass of 8.0 Gy whole-body irradiated mice treated with vehicle (O), 10 µmol (●), 20 µmol (■), or 40 µmol (▲) Cu(II)(5-DESS)/kg of body mass. Standard error bars have been omitted for clarity.

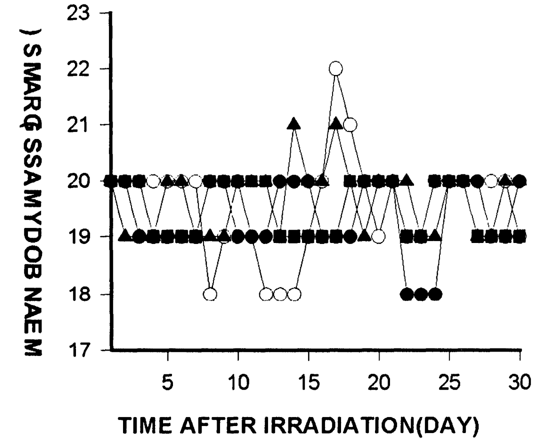

Figure 5. Mean body mass of 8.0 Gy whole-body irradiated mice treated with vehicle (O), 40 µmol (●), 60 µmol (■), or 80 µmol (▲) Cu(II)(5-DESS)/kg of body mass. Standard error bars have been omitted for clarity.

DISCUSSION

Doses of Cu(II)(5-DESS) used in this study ranged from 10 to 120µmol/kg of body mass or 0.2 to 2.4 µmol/mouse. All of these doses were non-toxic as evidenced by the lack of acute toxicity associated death in complex-treated groups prior to deaths occurring in vehicle-treated groups.

Since Cu(II)(5-DESS) increases survival when given prior to irradiation to facilitate physiological and biochemical responses following radiation injury, it is termed a radioprotective agent [2]. It seems improbable that radioprotectant activities of this complex are exclusively due to its superoxide dismutase-mimetic activity. Doses used in treatment would appear to be insufficient to be propitiously distributed to all tissues of 15 to 20 gm mice and enable increased survival. Distribution to key hematopoietic tissues may allow recovery of immune responses via de novo synthesis of Cu-dependent enzymes [12], including Cu-dependent SOD, and survival.

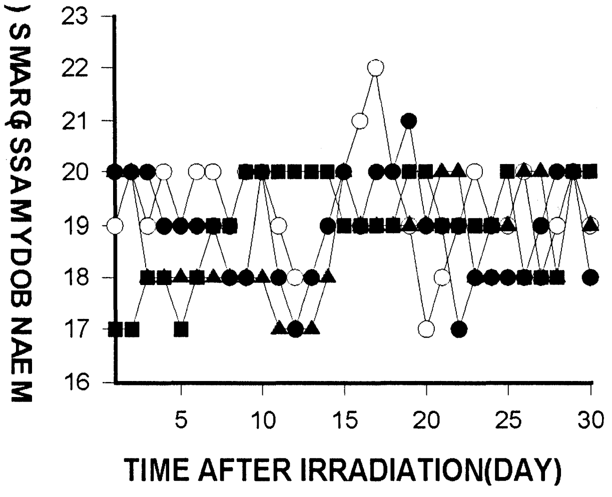

Figure 6. Mean body mass of 8.0 Gy whole-body irradiated mice treated with vehicle (O), 80 μmol (●), 100 μmol (g), or 120 μmol (▲) Cu(II)(5-DESS)/kg of body mass. Standard error bars have been omitted for clarity.

Studies by Soderburg et al. [15-17] have demonstrated that $Cu(II)_2(3,5\text{-}DIPS)_4$, which has both radioprotectant and radiorecovery activity, causes a rapid increase in recovery of immunocompetency when given either before or after irradiation. This recovery of immunocompetency is suggested to be due to facilitation of copper-dependent enzymes that have a role in maintaining immunocompetency. It is likely that radioprotectant activities of Cu(II)(5-DESS) are due to the ability of the complex to facilitate de novo synthesis of copper-dependent enzymes [12] required for rapid recovery of immunocompetency.

In recent years, there has been considerable interest in the role of copper in overcoming inflammatory disease states and facilitating tissue repair. One of the earliest (1940) uses of a copper compound as an anti-inflammatory drug was the use of allocupreide sodium as an anti-arthritic agent [18].

Subsequently, many copper complexes including salicylates have been found to have a variety of anti-inflammatory activities including: anti-polyarthritic, anti-ulcer, anti-convulsant, anti-cancer, analgesic, anti-diabetic, anti-carcinogenic, anti-mutagenic, anti-inflammatory bowel syndrome, prevention of ischemia-reperfusion injury, radioprotectant, and radiorecovery activites [18-20]. Since whole-body radiation injury is a model of whole-body inflammatory disease, the demonstration of radioprotectant activity for Cu(II)(5-DESS) is a demonstration of anti-inflammatory activity for this complex. Cu(II)(5-DESS) may also have other anti-inflammatory activities as well.

Studies of biochemical systems that mechanistically account for these antiinflammatory activities have revieled that copper complexes modulate: lysyl oxidases, eicosanoid syntheses and arachidonate metabolism, $Cu_2Zn_2$ SODs and demonstrate SOD-mimetic activity, stabilization of synovial and polymorphonuclear leukocyte lysosomal membranes, histaminic activity, lymphocyte mitogenic and chemotactic responses, stabilization of gamma-gobulin, GSH status, GSH-S-transferase, preprothrombin carboxylase, cytochrome P-450 monooxygenases, and/or NADPH-dependent P-450 reductases, 2-oxoglutarate-dependent hydroxylase, lipid peroxidation, angiogensis, and luteinizing hormone-releasing hormone secretion [18 and citations therein] as well as down-regulate nitric oxide synthase [21].

ACKNOWLEDGEMENTS

We are indebted to the National Cancer Institute for a Summer Student Fellowship (V.A.P. and M.A.W.), PHS grant number CA49425, the National Institute for General Medical Sciences, PHS grant number S06-RR08211 for Student Fellowships (E.W., J.A.K., III and H.S., III), the National Institutes of Health for two Research Apprenticeships (B.L.B. K.M., C.C. and T.B.), PHS grant number 5R25RR10281-03 and NASA Space Grant Consortium Student scholarship NL-4014q (H.J.I.).

REFERENCES

[1] T.D. Henderson, R.D. Henderson, H.J. Irving, W.M. Willingham, J.R.J. Sorenson, *Inflammopharmacol.* **3** (1995) 241.
[2] J.R.J. Sorenson, *Radiat. Res.* **132**, (1992) 19.
[3] W.M. Dale, L.H. Gray, W.J. Meredith, *Phil. Trans. Roy. Soc.* **242A**, (1949) 33.

[4] T.R. Sweeney, *A Survey of Compounds from the Anti-Radiation Drug Development Program of the U. S. Army Medical Research and Development Command.* Walter Reed Army Institute of Research, Washington D. C., September 1979.
[5] W.O. Foye, In *Burgers Medicinal Chemistry* (M.E. Wolf, ed.), Chapter 37, (1981) 11.
[6] M.M. Klingerman, A.T. Turrisi, L. Norfleet, M. Gramkowski, *First Conference on Radioprotectors and Anti-Carcinogens,* National Bureau of Standards, Gaithersburg, MD, June, 1982.
[7] A. Petkau, *Photochem. Photobiol.* **28**, (1978) 765.
[8] L.W. Oberley, A.L. Lindergren, S.A. Baker, R.H. Stevens, *Radiat. Res.* **68**, (1976) 320.
[9] H.P. Misra , I. Fridovich, *Arch. Biochem. Biophys.* **176**, (1976) 577.
[10] J. Schubert, *Copper and Peroxides in Radiology and Medicine.*Charles C. Thomas, Springfield, Illinois, 1964.
[11] J.R.J. Sorenson, *J. Med. Chem.* **27**, (1984) 1747.
[12] J.R.J. Sorenson, L.S.F. Soderberg, L.W. Chang, *Proced.Soc. Exptl. Biol. Med.* **210**: (1995) 191.
[13] D. Suriyachan, S. Patarakitvanit, P. Ratananakorn, *R. Thai Army Med. J.* **42**, (1989) 145.
[14] L. Steel, S. Seneviratne, W.E. Jackson, III, In *Anticarcinogens and Radiation Protection* (P. Cerutti, O.F. Nygaard, and M.G. Simic, Eds.), Plenum Press, New York, (1988) 355.
[15] L.S.F. Soderberg, J.B. Barnett, M.L. Baker, H. Salari, J.R.J. Sorenson, *Expt. Hematol.* **16**, (1988) 577.
[16] L.S.F. Soderberg, J.B. Barnett, M.L. Baker, H. Salari, J.R.J. Sorenson, *Expt. Hematol.* **18**, (1990) 801.
[17] L.S.F. Soderberg, J.B. Barnett, M.L. Baker, H. Salari, J.R.J. Sorenson, *Scand. J. Immunol.* **26**, (1987) 495.
[18] J.R.J. Sorenson, *Prog. Med. Chem.* **26**, (1989) 437.
[19] J.R.J. Sorenson, in "*Handbook of Metal-Ligand Interactions in Biological Fluids*: *Bioinorganic Medicine*" (G. Berthon, Ed.). Marcel Dekker, New York, **2** (1995) 1128.
[20] J.R.J. Sorenson, in "*Handbook of Metal-Ligand Interaction in Biological Fluids*: *Bioinorganic Medicine*" (G. Berthon, Ed.). Marcel Dekker, New York, **2** (1995) 1318.
[21] J. G. L. Baquial, J.R.J. Sorenson, *J. Inorg. Biochem.* **60** (1995)133.

**Received: March 17, 1999 - Accepted: March 31, 1999 - Received in revised camera-ready format: April 1, 1999**

